# High coffee consumption may increase aortic diameter and risk of abdominal aortic aneurysm in smokers

**DOI:** 10.1038/s41598-025-12668-2

**Published:** 2025-08-09

**Authors:** Joanna Kaluza, Otto Stackelberg, Martin Björck, Alicja Wolk

**Affiliations:** 1 Unit of Cardiovascular and Nutritional Epidemiology, Institute of Environmental Medicine, Karolinska Institutet, 171 77 Stockholm, Sweden; 2 Department of Human Nutrition, Warsaw University of Life Sciences– SGGW, Warsaw, Poland; 3 Section of Vascular Surgery, Department of Clinical Science and Education, Karolinska Institutet at Södersjukhuset, Stockholm, Sweden; 4 Department of Surgical Sciences, Section of Vascular Surgery, Uppsala University, Uppsala, Sweden; 5 Department of Surgical Sciences, Uppsala University, Uppsala, Sweden

**Keywords:** Abdominal aortic aneurysm, Consumption, Coffee, Prospective study, Smoking, Cardiovascular biology, Lifestyle modification, Risk factors

## Abstract

An association of coffee consumption with a risk of abdominal aortic aneurysm (AAA) is unknown. We hypothesized that coffee consumption influences aortic diameter and AAA risk, with smoking status as a modifier. The study included 42,723 Swedish men and 34,921 women (age 45–83 years) with infrarenal aortic diameter (IAD) measured in 8,109 men. Over 18.7 years, 1863 AAA cases (1585 non-ruptured, 278 ruptured) were identified. Among participants with coffee consumption ≤ 5 cups/day, current smokers versus never smokers had a 3-fold higher risk of non-ruptured and ruptured AAA (HR = 3.12, 95%CI = 2.62–3.71 and HR = 2.90, 95%CI = 1.95–4.31, respectively); the risk increased with coffee consumption > 5 cups/day and was a 4-fold higher (HR = 3.89, 95%CI = 3.12–4.85) for non-ruptured and a 4.6-fold higher (HR = 4.61, 95%CI = 2.72–7.86) for ruptured AAA (*P*-value- multiplicative-interaction = 0.009). 160 (2.0%) screened men had an IAD ≥ 30 mm. In men drinking daily ≤ 3 cups of coffee, current smokers versus never smokers had a 4-fold (OR = 4.09, 95%CI = 1.81–9.22) higher risk of IAD ≥ 30 mm; in men with higher coffee consumption (> 3 cups/day), the risk increased 6.6-fold (OR = 6.58, 95%CI = 2.98–14.6). In ex-smokers, the corresponding ORs were 1.67 (95%CI = 0.62–4.49) and 3.27 (95%CI = 1.27–8.40), respectively. In conclusion, high coffee consumption may increase risk of AAA and infrarenal aortic diameter in smokers.

## Introduction

Although coffee consumption is very common in many countries^1,2^and its association with multiple health outcomes has been extensively studied^3^until now, no study has examined coffee intake in relation to the risk of increased aortic diameter and risk of abdominal aortic aneurysm (AAA).

The European Society for Vascular Surgery 2019 Clinical Practice Guidelines on the Management of Abdominal Aorto-iliac Artery Aneurysms has emphasized that smoking is the strongest risk factor for AAA, with an odds ratio (OR) above 3^4^. Furthermore, studies indicate that current smokers consume more coffee compared to never smokers^5,6^.

Therefore, to fill the gap in knowledge on coffee consumption in relation to AAA incidence, we aimed to examine the association between coffee consumption and the risk of AAA in two large population-based cohorts of Swedish adults. Furthermore, we aimed to study the association of coffee consumption with the infrarenal aortic diameter (IAD) in a subgroup of screened men. Moreover, we aimed to examine whether smoking status, the main risk factor for AAA, modified the associations.

## Materials and methods

### Study population

The study included two population-based prospective cohorts: the Cohort of Swedish Men (COSM) and the Swedish Mammography Cohort (SMC). The COSM was established in 1997 when all men born in 1918–1952 residing in central Swedish counties (Västmanland and Örebro) were invited to join the cohort. The SMC was established in 1987–1990 when all women born in 1914–1949, who lived in Västmanland and Uppsala counties, were invited to a mammography-screening program.

In late fall 1997, 48,850 men (49% response rate) and 39,227 women (70% response rate) completed a 96-item food frequency questionnaire (FFQ) and answered questions about lifestyle factors (the questionnaires are available: https://www.simpler4health.se/researchers/questionnaires/).

**Fig. 1:**
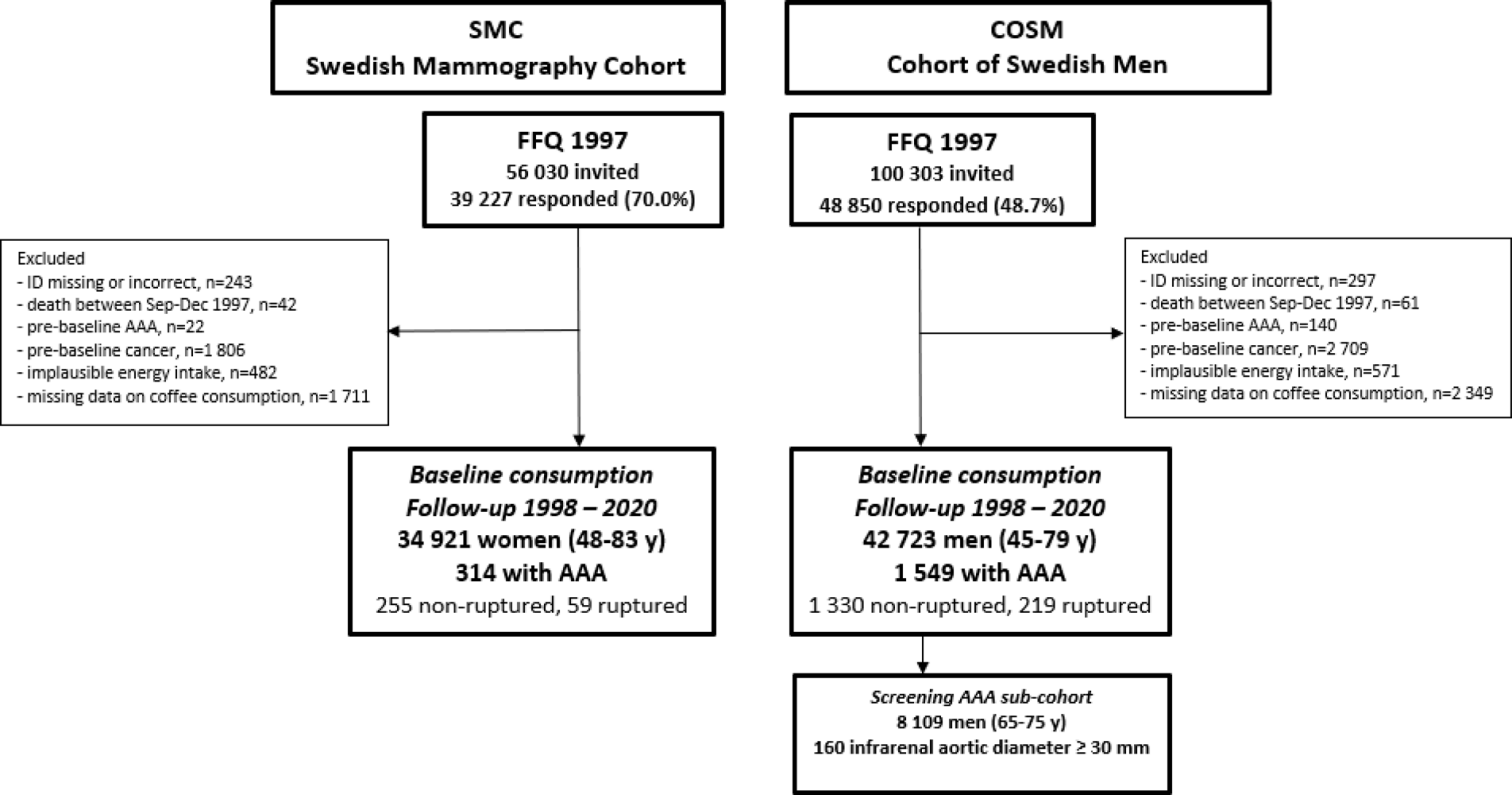
Flow-chart of the Swedish Mammography Cohort (SMC) and the Cohort of Swedish Men (COSM). Abbreviations: AAA, abdominal aortic aneurysm; FFQ, food frequency questionnaire.

Of the 48,850 men and 39,227 women who completed the questionnaire in 1997, we excluded participants with an incorrect or missing personal identity number, participants who died before the start of follow-up (January 1, 1998), and those with pre-baseline AAA diagnosis – Fig. 1. Moreover, due to the tendency to adopt healthier lifestyle behaviors after a cancer diagnosis (including dietary changes) and the simultaneous presence of an increased risk of death and cardiovascular disease (CVD) incidence, participants with a pre-baseline diagnosis of cancers other than non-melanoma skin cancer were excluded. Additionally, participants with implausible energy intake (± 3 SDs from the mean value of the log_e_-transformed) and those with missing data about coffee consumption were also excluded. After these exclusions, 42,723 men and 34,921 women were left for analysis.

Participants were followed from baseline (January 1, 1998), to the date of diagnosis of AAA or AAA repair, death, or the end of follow-up (December 31, 2020), whichever occurred first.

### Assessment of coffee consumption and covariates

The validated 96-item FFQ was used to collect data on coffee and other foods consumption in 1997^7^. Using an open-ended question, participants were asked how often daily, on average, during the last year, they consumed coffee, tea, and how many spoons of sugar they used. Based on one-week weighted diet records, a cup of coffee was defined from 160 to 210 ml (depending on the age and gender of participants). Energy intake was calculated by multiplying the frequency of food consumption by age-specific and gender-specific portion sizes and by the energy content of each food item^8^. Adherence to the Mediterranean diet was assessed using a previously developed modified Mediterranean diet (mMED) score^9,10^. The mMED score ranged from 0 to 8 points, and 0 points meaning the lowest adherence to the Mediterranean diet, while 8 points the highest adherence. In a validation study including 129 women from the SMC, comparing the FFQ to four one-week weighted diet records the Spearman correlation coefficient for coffee consumption was 0.63 [Wolk A, unpublished data].

Information about education level, body height and weight, time spent on walking and/or cycling, aspirin use, history of hypercholesterolemia, and family history of myocardial infarction (< 60 years old) was collected using the questionnaire. Participants were also asked about smoking status, age when they started smoking, the average number of cigarettes smoked per day through specified age categories (15–20, 21–30, 31–40, 41–50, and 51–60 years old and in current age), and if applicable about age of quit. Pack-years of smoking were calculated by multiplying the number of years of smoking by the reported number of cigarettes smoked per day within specific age categories. Data about history of diabetes was collected through linkage of participants’ ID with the National Diabetes Register and with the National Patient Register (ICD-10: E10-E14). Data about pre-baseline cardiovascular diseases (CVD) included cases of ischaemic heart disease (ICD-10: I20-I25), heart failure (I50, I110), stroke (I60, I61, I63, I64), and atrial fibrillation (I48) was collected via linkage with the National Patient Register. Data about history of hypertension was collected through linkage with the National Patient Register (I109) and complemented with self-reported data.

### Ascertainment of AAA

Incident cases of AAA, AAA repair, and death due to AAA were identified via linkage of the participants with the Swedish Inpatient Register and the Swedish National Cause of Death Register. We used the International Classification of Diseases and Related Health Problems − 10th Revision (ICD-10) to identify non-ruptured and ruptured aneurysm in the abdominal aorta (I71.4 and I71.3, respectively). AAA repair was identified through the Swedish Inpatient Register by use of the NOMESCO Classification of Surgical Procedures, and the Swedish National Registry for Vascular Surgery (Swedvasc) by use of the integrated AAA module in that registry. The Swedish Inpatient Register has no specific validity assessment for AAA; however, in general it has a high validity^11^ and nearly complete hospitalization data for the Swedish population since 1987^12,13^. Surgical procedures have been reported as incorrect in 2%, and missing in 5.3% of the records^11^and in an independent international validation, the external validity was 98.8% for AAA repair^13^.

### AAA screening sub-cohort

An analysis of a sub-cohort of 8109 men from the COSM who were screened for AAA during follow-up as a part of the national screening program (65–75 years old), using an infrarenal aortic diameter (IAD) ≥ 30 mm as outcome^14,15^ was performed.

### Statistical analysis

Cox proportional hazards regression models were used to estimate hazard ratios (HRs) with 95% confidence intervals (CIs) of total AAA and separately of non-ruptured and ruptured AAA. Coffee consumption was categorized as follows: ≤ 1.0 (reference), 1.1-3.0, 3.1-5.0, or > 5.0 cups/day. Model 1 was adjusted for age at the study baseline, sex, education, pack-years of smoking, and smoking cessation. Model 2 included the adjustments in Model 1, with additional adjustments for walking/cycling, BMI, aspirin use, history of diabetes, hypertension, hypercholesterolemia, CVD, family history of myocardial infarction, and tea consumption, sugar consumption, energy intake, and mMED score. To keep all participants in analyses missing data on educational level (0.5%), smoking status (5.4%), walking/cycling (8.7%), BMI (3.6%) and aspirin use (10.7%) were included in the models as separate categories. All covariates were prespecified and included in the models because they are known risk factors of AAA or potentially related to AAA and coffee consumption.

We have tested the assumption of proportional hazard using regression scaled Schoenfeld residuals against survival time, and no evidence of departure from the assumption was found. Using a likelihood ratio test, the interactions on the multiplicative scale between categories of coffee consumption and sex and smoking status were tested. Moreover, relative risks due to interaction (RERI) and attributable proportion of interaction (AP) were used to assess the additive interaction. Using a restricted cubic spline regression analysis with three knots (at the 10th, 50th, and 90th percentiles), the shape of associations between coffee consumption and risk of non-ruptured and ruptured AAA was examined. Linear trends were estimated by including daily cups of coffee consumption in the models as a continuous variable. Moreover, absolute risk difference (ARD), number needed to harm (NNH), and population attributable fraction (PAF) were calculated to further quantify the impact of coffee consumption on AAA. Additionally, we conducted mediation analyses using *med4way* Stata command to investigate the direct effect of coffee consumption on total AAA and the indirect effect mediated by smoking^16,17^.

An association between coffee consumption and IAD (< 30, ≥ 30 mm) was assessed using a multivariable logistic regression model in the screening sub-cohort of the COSM. The multivariable odds ratios (ORs) were adjusted for the same confounders as the Cox proportional hazard models except for the age, where age at screening instead of this at the study baseline was included.

All statistical analyses were performed using Stata software, version 17 (StataCorp, College Station, TX). Reported *P*-values are two-sided and the values ≤ 0.05 were generally considered statistically significant. To account for multiple tests, Bonferroni correction was applied for *P*-values.

## Results

### Coffee consumption and participant characteristics

A high percentage of men and women were coffee drinkers 98.9% and 98.7%, respectively, with the mean daily frequency of coffee consumption 3.5 ± 2.1 and 3.1 ± 1.8 cups, respectively. Participants in the highest versus those in the lowest category of coffee consumption (> 5.0 vs. ≤ 1.0 cups/day) were more likely to be men and current smokers, and less likely to have university education (Table 1). Moreover, with increasing coffee consumption, increased energy intake and sugar consumption, as well as decreased tea consumption were noted.

### Coffee consumption and AAA risk

**Fig. 2:**
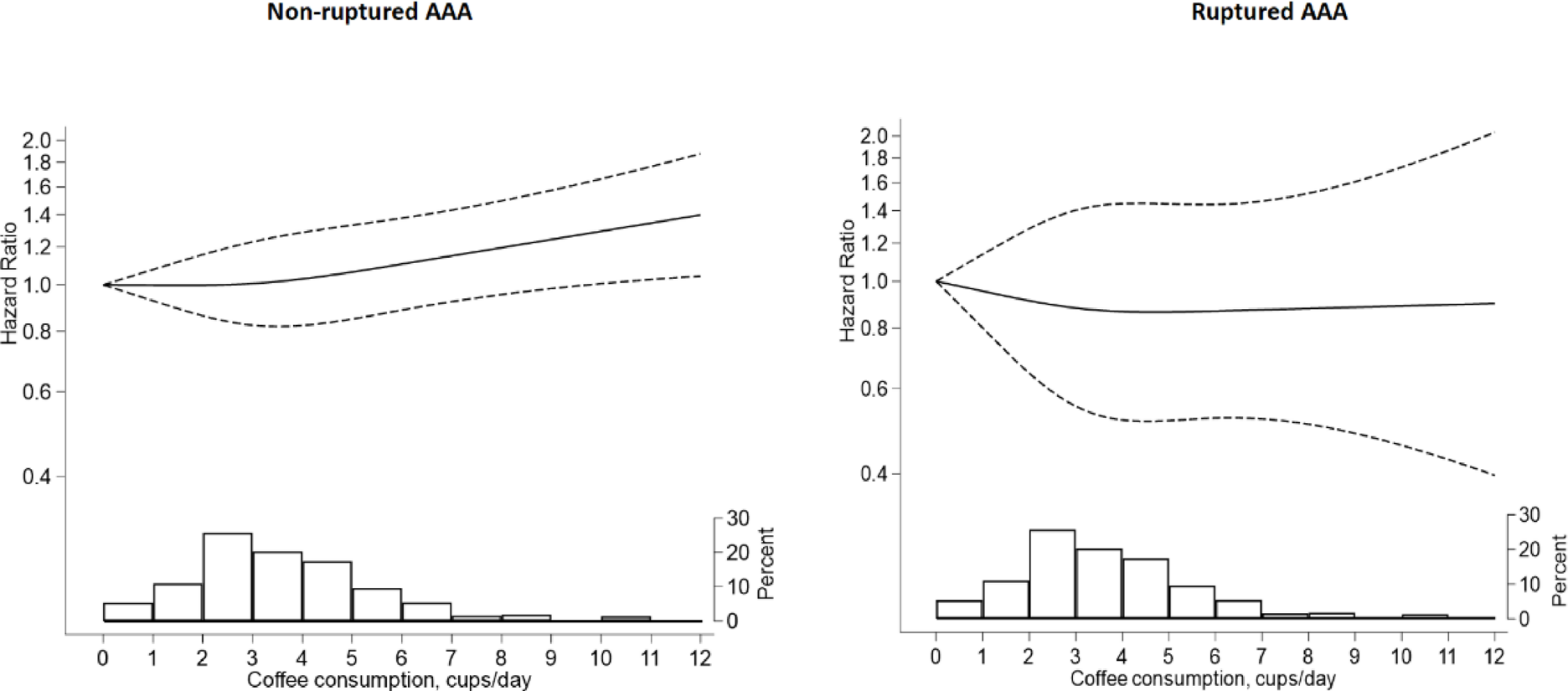
Multivariable adjusted hazard ratios (HR) of non-ruptured and ruptured abdominal aortic aneurysm (AAA) incidence as a function of coffee consumption in the Cohort of Swedish Men (*n* = 42,723) and in the Swedish Mammography Cohort (*n* = 34,921. The solid curve represents the restricted cubic spline, and dashed-dotted lines represent 95% confidence intervals (CI). The distribution of coffee consumption is presented at the bottom of each figure as a histogram. HR and 95% CI were adjusted for age at the study baseline, sex, education (primary, high school, or university), pack-years of smoking (years, continuous), and smoking cessation (years, continuous), body mass index (< 18.5, 18.5-24.9, 25-29.9, or ≥ 30 kg/m^2^), walking/cycling (< 20, 20–40, 40–60, or > 60 min/day), history of diabetes (yes, no), hypertension (yes, no), hypercholesterolemia (yes, no), cardiovascular diseases (yes, no), family history of myocardial infarction (yes, no), aspirin use (yes, no), tea consumption (0, 0.1–0.9, 1.0-1.9, or ≥ 2 servings/day), sugar intake (0, 0.1-1, 1.1-3, 3.1-5, or > 5 servings/day) and intake of energy (kcal/day, continuous). Missing data on educational level (0.5%), body mass index (3.6%), walking/cycling (8.7%), and aspirin use (10.7%) were included in the models as separate categories.

During an average of 18.7 ± 6.2 years of follow-up from 1998 to 2020 (1,448,393 person-years), 1863 incident AAA cases were identified, of which 1585 (85.1%) were non-ruptured and 278 (14.9%) ruptured. There was no interaction with gender (*P*-value for interaction = 0.57); therefore, we analyzed men and women together. Among participants in the highest coffee consumption category (> 5 cups/day), the ARD per 10,000 person-years was 8.2 for total AAA and 8.0 for non-ruptured AAA compared to participants in the lowest category (≤ 1 cap/day). This correspondents to 1,224 and 1243 person-years needing to be exposed for one additional case of total AAA and non-ruptured AAA, respectively. Participants with coffee consumption > 5 cups/day had a higher risk of total and non-ruptured AAA incidence compared to those with consumption ≤ 1 cup/day (HR = 1.22, 95%CI = 1.01–1.48 and HR = 1.25, 95%CI = 1.02–1.54, respectively), with *P*-trend below Bonferroni correction threshold – Table 2. Among participants with coffee consumption > 5 cups/day, the proportion of total AAA and non-ruptured AAA cases due to this exposure was 12.3% and 14.1%, respectively. No similar association was observed for ruptured AAA (HR = 1.03, 95%CI = 0.62–1.71); however, the limited number of ruptured cases led to low statistical power and a wide 95% confidence interval. The shape of the associations between coffee consumption and risk of non-ruptured AAA and ruptured AAA is presented in Fig. 2.

### AAA screening sub-cohort

In the sub-cohort of 8109 men with available measurements of IAD, 160 (2.0%) of men were classified having AAA. Participants with 3.1-5 and > 5 cups/day versus those with ≤ 3 cups/day coffee consumption had increased risk of IAD ≥ 30 mm (OR = 1.72, 95%CI = 1.16–2.55 and OR = 1.86, 95%CI = 1.19–2.92, respectively) with the linear trend between coffee consumption and IAD – Table 2. Each 1-cup increment in coffee consumption was associated with a 8% (95%CI = 1–17%, *P*-linear-trend = 0.03) increased risk of IAD ≥ 30 mm.

**Table 1:** Age-standardized baseline characteristics of 42,723 men (1,549 with AAA) from the Cohort of Swedish Men and 34,921 women (314 with AAA) from the Swedish Mammography Cohort by categories of coffee consumption, baseline 1998.

	Coffee consumption, range (median) cups/day				*P*-value^a^
	≤ 1.0 (1.0)	1.1-3.0 (2.1)	3.1-5.0 (4.0)	> 5.0 (6.0)	
No. of people	9,480	36,925	22,045	9,194	
Total no. with AAA	194	795	532	342	< 0.001
Total no. with ruptured AAA	34	138	69	37	0.57
Men, n (%)	5028 (53.3)	18,782 (51.1)	12,761 (57.5)	6152 (65.5)	< 0.001
Age at baseline, years, means (SD)	62.0 (9.6)	61.8 (9.6)	60.0 (9.3)	58.0 (8.8)	< 0.001
University education, n (%)^b^	2014 (22.6)	6682 (19.0)	3426 (14.8)	1218 (11.7)	< 0.001
Smoking status, n (%)^b^ current smokers * pack-years*,* median* ex-smokers * pack-years*,* median* never	1470 (15.9) *15.8* 2986 (32.1) *11.0* 4872 (52.0)	7203 (20.0) *17.1* 11 502 (31.9) *10.5* 17 664 (48.1)	6095 (27.6) *20.8* 7274 (33.2) *12.6* 8358 (39.2)	3854 (40.4) *25.2* 2730 (29.8) *14.7* 2450 (29.8)	< 0.001
BMI, kg/m^2^, means (SD)b	25.4 (3.9)	25.3 (3.6)	25.5 (3.6)	25.7 (3.7)	< 0.001
Walking/cycling (min/day), n (%)^b^ <20 20–40 40–60 >60	3029 (35.1) 2698 (31.3) 1423 (16.4) 1525 (17.2)	11 205 (33.3) 11 001 (32.7) 5866 (17.2) 5858 (16.8)	6862 (34.0) 6449 (31.8) 3361 (16.7) 3497 (17.5)	3225 (38.3) 2336 (27.8) 1241 (15.3) 1461 (18.6)	< 0.001
Diabetes, n (%)	874 (8.7)	2898 (7.5)	1520 (7.3)	686 (8.9)	< 0.001
Hypertension, n (%)	2608 (26.4)	9138 (24.0)	4725 (22.2)	1775 (21.5)	< 0.001
Hypercholesterolemia, n (%)	1248(12.9)	4731 (12.6)	2811 (12.8)	1250 (14.0)	0.16
Cardiovascular diseases, n (%)^c^	1333 (12.9)	4233 (10.7)	2048 (10.1)	786 (10.7)	< 0.001
Family history of myocardial infraction, n (%)	1172 (12.5)	4427 (12.1)	2714 (12.2)	1254 (13.0)	< 0.001
Aspirin use, n (%)^b^	3756 (43.8)	14 288 (43.3)	8343 (42.6)	3477 (42.6)	< 0.001
Energy intake, kcal/day, means (SD)	2107 (815)	2151 (782)	2347 (851)	2563 (957)	0.001
Modified MED score, points, means (SD)	3.6 (1.7)	3.7 (1.6)	3.7 (1.6)	3.6 (1.7)	0.06
Tea consumption, servings/day, means (SD)	1.2 (1.5)	0.6 (1.0)	0.4 (0.8)	0.3 (0.8)	< 0.001
Sugar in tea/coffee, servings/day, means (SD)	1.1 (1.8)	1.1 (1.9)	1.6 (2.7)	2.1 (3.6)	< 0.001

Abbreviations: AAA, abdominal aortic aneurysm; BMI, body mass index; MED, Mediterranean Diet; SD, standard deviation.

^a^*P* values were calculated across categories of coffee consumption by using age-adjusted linear models for continuous variables and Pearson’s Chi-square for categorized variables. ^b^Missing data on educational level (0.5%), smoking status (5.4%), BMI (3.6%), walking/cycling (8.7%), and aspirin use (10.7%) were included in the models as separate categories; percentages for these variables are based on the dataset with complete data. ^c^Cardiovascular diseases included ischemic heart disease (ICD10: I20-I25), heart failure (I50, I110), stroke (I60, I61, I63, I64), and atrial fibrillation (I48).

**Table 2:** Association of coffee consumption with risk of abdominal aortic aneurysm (AAA) in 42,723 men (1,549 with AAA) from the Cohort of Swedish Men and in 34,921 women (314 with AAA) from the Swedish Mammography Cohort, follow-up 1998 to 2020.

		Coffee consumption, range (median) cups/day								*P*-value
		≤ 1.0 (0.6)			1.1-3.0 (2.1)	3.1-5.0 (4.0)		> 5.0 (6.0)		
Total AAA										
No. of cases/Person-years		194/171,171		795/683,507		532/418,369			342/175,346	
Model 1, HR (95% CI)^a^		1.00 (Ref.)		1.05 (0.89–1.22)		1.04 (0.88–1.23)			1.37 (1.15–1.64)	0.001
Model 2, HR (95% CI)^b^		1.00 (Ref.)		1.00 (0.85–1.17)		0.96 (0.81–1.14)			1.22 (1.01–1.48)	0.021
**Non-ruptured AAA**										
No. of cases/Person-years		160/171,171		657/683,507		463/418,369			305/175,346	
Model 1, HR (95% CI)^a^		1.00 (Ref.)		1.04 (0.88–1.24)		1.07 (0.89–1.28)			1.38 (1.15–1.69)	< 0.001^f^
Model 2, HR (95% CI)^b^		1.00 (Ref.)		1.00 (0.84–1.19)		1.00 (0.82–1.20)			1.25 (1.02–1.54)	0.012^f^
***Infrarenal aortic diameter (IAD) ≥ 30 mm*** ^c^										
No. of cases/Participants		11/861		44/3,460		58/2,531			47/1,257	
Model 2, OR (95% CI)^d^		1.00 (Ref.)		0.91 (0.46–1.82)		1.59 (0.80–3.18)			1.73 (0.83–3.58)	0.047
Model 2, OR (95% CI)^d^		1.00 (Ref.)^e^				1.72 (1.16–2.55)			1.86 (1.19–2.92)	0.047
**Ruptured AAA**										
No. of cases/Person-years		34/171,171		138/683,507		69/418,369			37/175,346	
Model 1, HR (95% CI)^a^		1.00 (Ref.)		1.06 (0.73–1.54)		0.90 (0.59–1.35)			1.20 (0.75–1.93)	0.819
Model 2, HR (95% CI)^b^		1.00 (Ref.)		0.99 (0.67–1.45)		0.81 (0.52–1.25)			1.03 (0.62–1.71)	0.719

Abbreviations: HR, hazard ratio; OR, odds ratio; 95% CI, 95% confidence interval.

^a^Adjusted for age at the study baseline (years, continuous), sex, pack-years of smoking (years, continuous), and smoking cessation (years, continuous). ^b^Adjusted as Model 1 and additional adjusted for education (primary, high school, or university), BMI (< 18.5, 18.5-24.9, 25-29.9, or ≥ 30 kg/m^2^), walking/cycling (< 20, 20–40, 40–60, or > 60 min/day), history of diabetes (yes, no), hypertension (yes, no), hypercholesterolemia (yes, no), and cardiovascular diseases (yes, no), family history of myocardial infarction (yes, no), aspirin use (yes, no), tea consumption (0, 0.1–0.9, 1.0-1.9, or ≥ 2 servings/day), sugar intake (0, 0.1-1, 1.1-3, 3.1-5, or > 5 servings/day), intake of energy (kcal/day, continuous), and modified Mediterranean Diet Score (points, continuous). Missing data on educational level (0.5%), smoking status (5.4%), BMI (3.6%), walking/cycling (8.7%), and aspirin use (10.7%) were included in the models as separate categories. ^c^Sub-cohort of 8,109 men who were screened for AAA during follow-up. ^d^Adjusted for age at screening; otherwise, it is like Model 2. ^e^The two first categories were combined due to a small number of cases. ^f^The *P*-value remained significant even after applying the Bonferroni correction.

### Associations by smoking status

The group with high coffee consumption (> 5 cups/day) were 2.5-fold more likely to be current smokers (40.4%) compared to those with low coffee consumption (≤ 1 cup/day; 15.9%), while the percentage of ex-smokers did not differ between the groups (29.8 vs. 32.9%, respectively). In current smokers, higher coffee consumption was associated with more pack-years smoked – the median of pack-years smoked in the group drinking > 5 vs. ≤ 1 cup/day was 25.2 and 15.8, respectively. A similar association was observed in ex-smokers where the median of pack-years smoked was 14.7 in the group with coffee consumption > 5 cups/day, respectively 11.0 in the group with a consumption ≤ 1 cup/day.

A statistically significant interaction between coffee consumption and smoking status in relation to total AAA incidence was found. The *P*-value for interaction on the multiplicative scale was 0.009 and remained statistically significant after Bonferroni correction. Interaction on the additive scale was RERI= −0.85, 95%CI: −1.71 to 0.02, and AP= −0.56, 95%CI: −1.13 to 0.01.

The total excess hazard ratio (eHR) for total AAA associated with consuming > 5 vs. ≤ 5 cups of coffee per day was 0.53 (95%CI = 0.32–0.73) (Table 3). This eHR was decomposed into four components: *(1)* the indirect effect, which was 0.13 (95%CI = 0.12–0.15); *(2)* the mediated interaction, which was 0.10 (95%CI = 0.05–0.14); *(3)* the reference interaction, which was 0.38 (95%CI = 0.21–0.55); and *(4)* the controlled direct effect (assuming a reference group of never smokers), which was − 0.08 (95%CI=−0.26-0.09). Among these components, the reference interaction was the largest contributor, highlighting the significant role of the interaction between coffee consumption and smoking status in relation to AAA risk. In contrast, the negative controlled direct effect – representing the effect of coffee consumption not mediated by smoking – suggests a slight, though non-significant, protective effect on the risk of total AAA.

**Table 3:** Total excess hazard ratio (eHR) and its attributable proportions of coffee consumption (> 5 vs. ≤ 5 cups/day) on total abdominal aortic aneurysm (AAA) due to mediation and interaction with smoking status.

Component	Excess Hazard Ratio^a^	(95% CI)^a^	*P*-value	Proportion attributable^a^	(95% CI)^a^
Total effect	0.53	(0.32 to 0.73)	< 0.001	100%	
Controlled direct effect	−0.08	(−0.26 to 0.09)	0.338	−16%	(−53–21%)
Reference interaction	0.38	(0.21 to 0.55)	< 0.001	72%	(46–98%)
Mediated interaction	0.10	(0.05 to 0.14)	< 0.001	19%	(12–25%)
Pure indirect effect	0.13	(0.12 to 0.15)	< 0.001	25%	(15–36%)

Abbreviations: 95% CI, 95% confidence interval.

^a^ Adjusted for age at the study baseline, sex, education (primary, high school, or university), body mass index (< 18.5, 18.5-24.9, 25-29.9, or ≥ 30 kg/m^2^), walking/cycling (< 20, 20–40, 40–60, or > 60 min/day), history of diabetes (yes, no), hypertension (yes, no), hypercholesterolemia (yes, no), cardiovascular diseases (yes, no), family history of myocardial infarction (yes, no), aspirin use (yes, no), tea consumption (0, 0.1–0.9, 1.0-1.9, or ≥ 2 servings/day), sugar intake (0, 0.1-1, 1.1-3, 3.1-5, or > 5 servings/day), and intake of energy (kcal/day, continuous). Missing data on educational level (0.5%), body mass index (3.6%), walking/cycling (8.7%), and aspirin use (10.7%) were included in the models as separate categories.

**Fig. 3:**
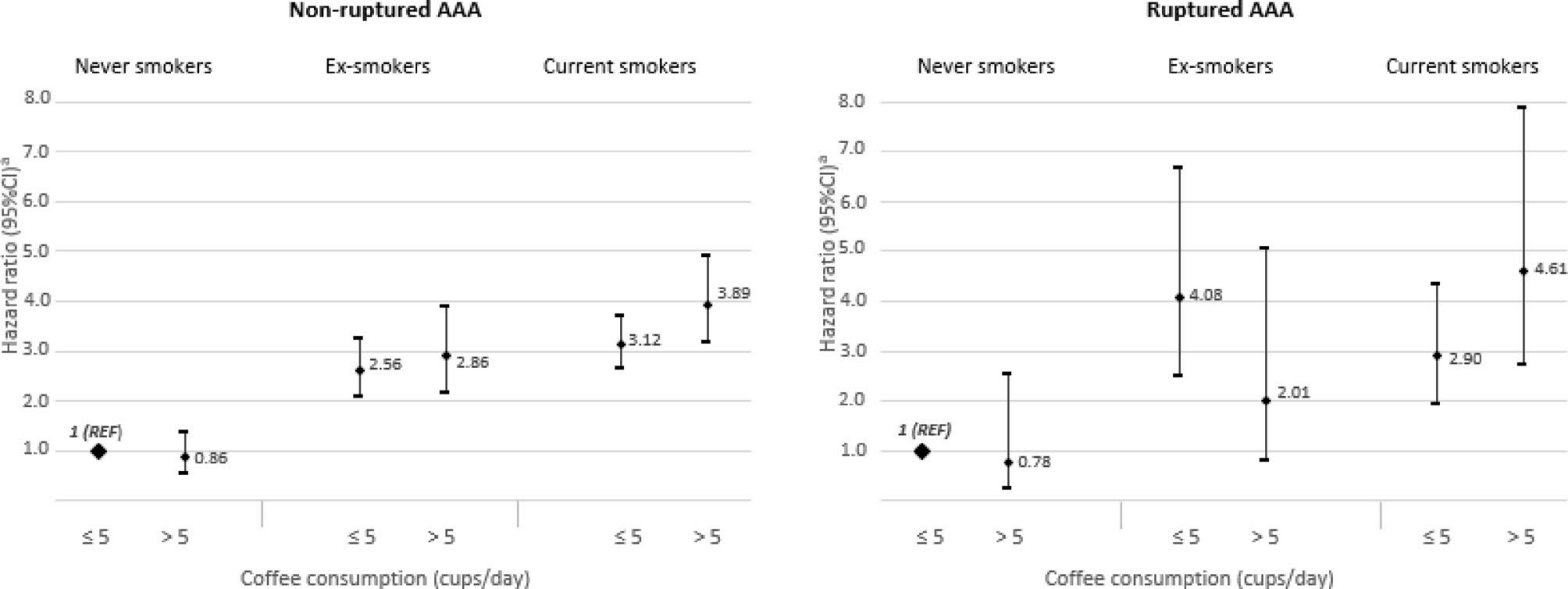
Combined association of coffee consumption and smoking status with risk of abdominal aortic aneurysm (AAA). ^a^ Adjusted for age at the study baseline, sex, education (primary, high school, or university), pack-years of smoking (years, continuous), smoking cessation (years, continuous), body mass index (< 18.5, 18.5-24.9, 25-29.9, or ≥ 30 kg/m^2^), walking/cycling (< 20, 20–40, 40–60, or > 60 min/day), history of diabetes (yes, no), hypertension (yes, no), hypercholesterolemia (yes, no), cardiovascular diseases (yes, no), family history of myocardial infarction (yes, no), aspirin use (yes, no), tea consumption (0, 0.1–0.9, 1.0-1.9, or ≥ 2 servings/day), sugar intake (0, 0.1-1, 1.1-3, 3.1-5, or > 5 servings/day) and intake of energy (kcal/day, continuous). Missing data on educational level (0.5%), body mass index (3.6%), walking/cycling (8.7%), and aspirin use (10.7%) were included in the models as separate categories.

To examine the combined effect of coffee consumption and smoking status in relation to the risk of AAA, we conducted an analysis including these variables in the same model with never smokers with coffee consumption ≤ 5 cups/day as a reference group (Fig. 3). Compared to the reference group, current smokers with coffee consumption ≤ 5 cups/day had a 3-fold higher risk of non-ruptured as well as ruptured AAA (HR = 3.12, 95%CI = 2.62–3.71 and HR = 2.90, 95%CI = 1.95–4.31, respectively). High coffee consumption seemed to modify the association between smoking and the risk of AAA; the risk increased with coffee consumption > 5 cups/day and compared to the reference group was 4-fold higher (HR = 3.89, 95%CI = 3.12–4.85) for non-ruptured and 4.6-fold higher (HR = 4.61, 95% CI = 2.71–7.86) for ruptured AAA. No association was observed between high compared to low coffee consumption (> 5 vs. ≤ 5 cups/day) and risk of non-ruptured and ruptured AAA in never smokers.

### AAA screening sub-cohort

**Fig. 4:**
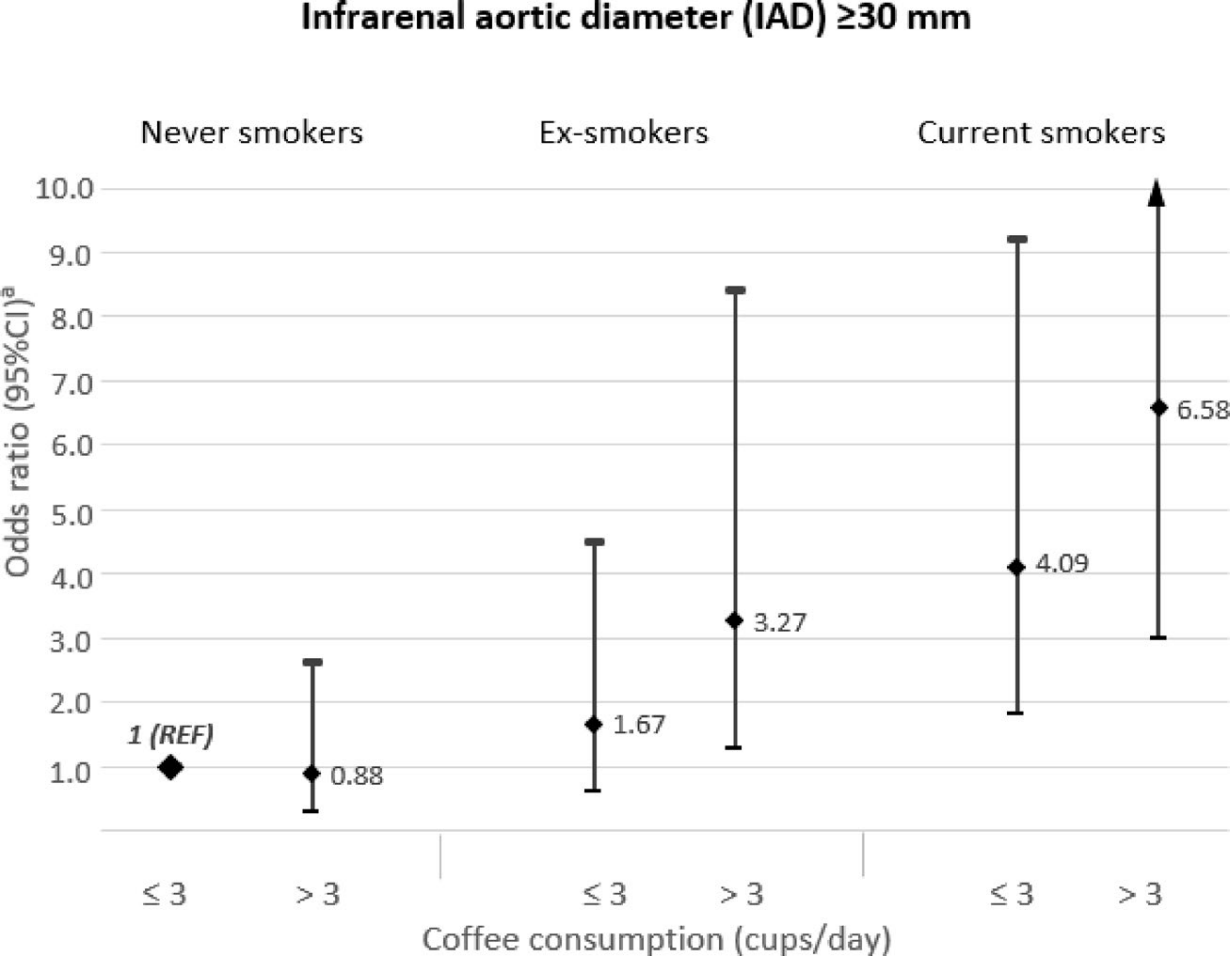
Combined association of coffee consumption and smoking status with risk of infrarenal aortic diameter (IAD) ≥ 30 mm. ^a^ Adjusted for age at the study baseline, sex, education (primary, high school, or university), pack-years of smoking (years, continuous), smoking cessation (years, continuous), body mass index (< 18.5, 18.5-24.9, 25-29.9, or ≥ 30 kg/m^2^), walking/cycling (< 20, 20–40, 40–60, or > 60 min/day), history of diabetes (yes, no), hypertension (yes, no), hypercholesterolemia (yes, no), cardiovascular diseases (yes, no), family history of myocardial infarction (yes, no), aspirin use (yes, no), tea consumption (0, 0.1–0.9, 1.0-1.9, or ≥ 2 servings/day), sugar intake (0, 0.1-1, 1.1-3, 3.1-5, or > 5 servings/day), intake of energy (kcal/day, continuous), and modified Mediterranean Diet Score (points, continuous). Missing data on educational level (0.5%), body mass index (3.6%), walking/cycling (8.7%), and aspirin use (10.7%) were included in the models as separate categories.

Results of the combined association of coffee consumption and smoking status in relation to the risk of an IAD ≥ 30 mm are presented in Fig. 4. Compared to the reference group (never smokers with coffee consumption ≤ 3 cups/day), current smokers men with coffee consumption ≤ 3 cups/day had a 4-fold higher risk of having an IAD ≥ 30 mm (HR = 4.09, 95%CI = 1.81–9.22), while the risk increased 6.6-fold when coffee consumption was > 3cups/day in current smokers (HR = 6.58, 95%CI = 2.98–14.56). Male ex-smokers with coffee consumption ≤ 3 and > 3 cups/day compared to the reference group had a 1.7-fold (HR = 1.67, 95%CI = 0.62–4.49) and 3.3-fold (HR = 3.27, 95%CI = 1.27–8.40), respectively, increased risk of having an IAD ≥ 30 mm. In men who never smoked, the risk of having an IAD ≥ 30 mm did not differ with coffee consumption > 3 cups/day compared to those with consumption ≤ 3 cups/day.

## Discussion

In these large population-based prospective cohorts of men and women, high coffee consumption (> 5 cups/day) was associated with a higher risk of AAA among smokers. In men screened for AAA, consumption of > 3 cups of coffee per day increased the risk of having an infrarenal aortic diameter ≥ 30 mm. Among participants who had never smoked, coffee consumption was not associated with the risk of AAA and did not impact infrarenal aortic diameter.

To the best of our knowledge, this is the first study to examine the association between coffee consumption and a risk of increased aortic diameter and risk of AAA. Until now, the potential health benefits of coffee consumption have been examined in prospective cohort studies, mostly in relation to CVD mortality and CVD events^3^. The results of an umbrella review of meta-analyses indicate that coffee consumption is associated with a non-linear decrease in risk of mortality from CVD causes and a non-linear decrease in risk of CVD incidence with the largest benefits at consumption of 3–4 cups per day^3^. However, in this umbrella meta-analyses review the associations were not stratified by smoking status. Until now, some studies on coffee consumption and CVD events have taken into consideration smoking status with inconsistent results^18–20^.

A potential mechanism of AAA development related to coffee drinking may be associated with a risk of increasing low-density lipoprotein (LDL) cholesterol levels^21^. Bioactive compounds present in coffee such as chlorogenic acid and diterpenes (mainly cafestol and kahweol), may raise serum LDL and very-low-density lipoprotein (VLDL) cholesterol levels by impairing the body’s ability to regulate cholesterol through their effects on bile acid metabolism^22^. These compounds also influence the expression of some genes involved in lipid metabolism, further affecting cholesterol levels^22^. Scandinavian boiled coffee, an unfiltered type of coffee, contains particularly high amounts of kahweol and cafestol (up to 100 and 80 times more, respectively). A Swedish study conducted in the same region found that 11% of participants in the case group with nonfatal myocardial infarction consumed boiled coffee, compared to 7% in a control group. When comparing individuals who drank boiled coffee to those who consumed filtered coffee, a higher incidence risk of nonfatal myocardial infarction was observed among those drinking boiled coffee (RR: 1.41; 95% CI: 1.07–1.80 in men and 1.63; 95% CI: 1.04–2.56 in women)^23^.

Cigarette smoking increases the risk of increased aortic diameter^24,25^ and the risk of AAA by inducing oxidative stress and inflammation^26,27^. It disturbs collagen synthesis, leading to a breakdown of arterial elastin and collagen^28,29^and promotes vascular smooth muscle cell dysfunction^30^. Results of a meta-analysis indicate that current and ex-smokers versus never smokers have a 4.9- and 2.1-fold higher risk of AAA, respectively^31^. In a previous study of the same cohorts, it was found that current smokers, men and women with more than 20 pack-years compared to never smokers, had a 6.6-fold and 11.0-fold, respectively, higher risk of AAA incidence^32^.

In our study, a statistically significant interaction between coffee consumption and smoking status in relation to AAA incidence was observed (*P*-value for interaction = 0.009). There are several potential mechanisms through which the combination of coffee consumption and smoking may increase the risk of AAA. Both smoking and caffeine independently contribute to increased aortic stiffness and wave reflections, which are key determinants of cardiovascular health. When combined, these effects are amplified, leading to a significant increase in pulse-wave velocity and augmentation index, which are indicators of arterial stiffness and cardiovascular risk^33,34^. This seems to be particularly important because of the fact that coffee consumption and cigarette smoking are strongly associated^6^. Results of a Mendelian randomization study indicate that higher cigarette smoking causally increases coffee intake; each additional cigarette smoked per day by current smokers was associated with higher coffee consumption (0.10 cups/day, 95%CI = 0.03–0.17)^6^. This result was consistent with the faster metabolism of caffeine by smokers. Each additional copy of the minor allele of rs16969968 (associated with a higher number of cigarettes smoked) was associated with higher coffee consumption (0.16 cups/day, 95%CI = 0.11–0.20)^6^.

Moreover, caffeine consumption may influence such behaviors as a temptation to smoke and increased satisfaction with cigarette taste. It may be associated with an increased urge to smoke and be affected by the memorial processing of smoking^35^. Moreover, the activity of CYP1A2 (the enzyme responsible for caffeine metabolizing) is increased by cigarette smoking. Polycyclic aromatic hydrocarbons present in tobacco smoke bind to the aryl hydrocarbon receptor, which also activates the CYP1A2 gene^36^. Thus, faster caffeine metabolism in smokers may lead to higher coffee consumption to experience its stimulating effects and avoid withdrawal. It may also allow an increase in caffeine consumption without symptoms of its toxicity^37^.

Strengths of the current study are the large population-based cohorts, prospective study design, the use of the validated FFQ to collect dietary data, and detailed information on potential confounders including smoking status. It allowed adjusting results for variables strongly associated with the risk of AAA development. Another strength of the study is the complete follow-up of the participants via linkage of both cohorts with the well-validated Swedish registers and the high number of AAA cases. The sub-study on 8109 men who underwent population-based AAA screening, among whom the aorta was measured, adds precision to the study.

Our study also has some limitations. First, the FFQ included only one question on total coffee consumption; thus, analyzing filtered versus unfiltered coffee consumption with a risk of AAA was impossible. Although the total coffee consumption had high validity^7^some misclassification in the consumption is inevitable. However, because of the prospective design of the study, such sources of misclassification would most likely attenuate potential associations. Second, although HRs were multivariable-adjusted, the possibility of residual dietary confounding and other unmeasured factors influencing the results cannot be ruled out. Additionally, in our study, covariates were assessed only once in 1997, requiring the assumption that lifestyle habits remained unchanged during the follow-up period. Third, the use of register-based asymptomatic cases may cause an under-detection of asymptomatic AAA in participants classified as non-diseased, which could have led to an underestimation of the risk estimates. However, when using measurements of aortic diameter as the outcome, in the sub-cohort that underwent screening with ultrasound, where there is virtually no risk of under-detection of AAA status, findings were similar to register-based outcomes. Fourth, despite the extensive follow-up period of over 18 years, covering 1,448,393 person-years, the number of ruptured AAA cases still was too low to achieve adequate statistical power. Therefore, the observed lack of association between coffee consumption and ruptured AAA should be interpreted with caution. Finally, the observational nature of the study limits the ability to infer causality or direct clinical implications from the findings.

## Conclusion

Obtained results indicate that high coffee consumption may be associated with increased aortic diameter and a higher risk of abdominal aortic aneurysm among ever smokers, but not among never smokers. However, due to potential confounding factors and the observational nature of the study, these results should be interpreted with caution. Further studies in diverse populations are needed before definitive public health recommendations can be made. The findings are important from a public health point of view but also shed further light on the complex pathophysiology of AAA disease.

## Data Availability

The datasets analysed during the current study are not publicly available due to institutional security procedures and database protection policies, but are available from the corresponding author (Joanna Kaluza) on reasonable request.
